# Supplementary material for: The intention to use Kangaroo Mother Care among first-time mothers 15-24 years in Kinshasa, DRC: An application of the integrated behavioral model
Source: PLOS Glob Public Health. 2026 Oct 23;6(10):e0006015. doi: 10.1371/journal.pgph.0006015 (PMC13693924; doi:10.1371/journal.pgph.0006015)
Supplement: Supplementary file 1 [file pgph.0006015.s001.docx]

**Supplemental Table 1. Operationalization of study variables**

| **Variable** | **Definition** | **Coding** |
| --- | --- | --- |
| **Outcome** |  |  |
| Intention to use KMC | How likely to use KMC in the future if they had a low-birth weight baby | 1-very likely; 0- likely, unlikely, very unlikely |
| **Attitudes** |  |  |
| Instrumental attitudes | Number of benefits of KMC a respondent could name | 0,1,2,3,4,5+ |
| Approval | Approve of KMC | 1-Yes, 0-No |
| **Perceived norms** |  |  |
| Descriptive norms | How many other FTMs with a preterm of low-birth weight baby practiced KMC in their community | 1-All, more than half, about half; 0-less than half, none |
| Normative expectations | Agreement with “Most people who are important to me think I ought to practice KMC if I have a low-birth weight baby” | Strongly agree (ref); agree; disagree/strongly disagree |
| **Personal agency** |  |  |
| Self-efficacy | General self-efficiency from the General Self-Efficacy Scale. Items assess respondents’ ability to overcome adversity and solve problems | Additive index ranging from 10-40; higher values correspond with greater self-efficiency |
| KMC autonomy | Would still practice KMC even if people most important to them did not want them to | 1-Yes; 0-No |
| **Covariates** |  |  |
| Marital status | If respondent had ever been married | 1-Yes; 0-No |
| Age | Age at baseline | 15-19 years (ref); 20-24 years |
| Education | Highest level of education completed at baseline | None/primary/secondary incomplete (ref); secondary complete or higher |
| Household wealth | Index constructed using principal components analysis of 19 indicators of housing, water, sanitation, and household assets | Low (ref); medium; high terciles |
| Employment status | If the respondent had worked, excluding housework, in the 12 months preceding the baseline survey | 1-Yes; 0-No |
| Previous knowledge of KMC | Respondent had heard of KMC at baseline | 1-Yes; 0-No |
| Residence in intervention or comparison health zone | Residence in a Momentum intervention or comparison health zone | 1-Intervention health zone; 0-Comparison health zone |
