# Supplementary material for: The intention to use Kangaroo Mother Care among first-time mothers 15-24 years in Kinshasa, DRC: An application of the integrated behavioral model
Source: PLOS Glob Public Health. 2026 Oct 23;6(10):e0006015. doi: 10.1371/journal.pgph.0006015 (PMC13693924; doi:10.1371/journal.pgph.0006015)
Supplement: Supplementary file 2 [file pgph.0006015.s002.docx]

**Supplemental Table 2. Logistic regression models of KMC intentions by age group, FTMs 15-24 years, Kinshasa, DRC 2020**

| **Variables** | **Model 3A** | | **Model 3B** | |
| --- | --- | --- | --- | --- |
|  | ***15-19 years*** | | ***20-24 years*** | |
|  | **Adjusted Odds Ratio** | **95% CI** | **Adjusted Odds Ratio** | **95% CI** |
| **Attitude** |  |  |  |  |
| Instrumental Attitude |  |  |  |  |
| No KMC benefits |  |  |  |  |
| 1 KMC benefit | 1.60 | (0.58-4.39) | 1.46 | (0.53-4.02) |
| 2 KMC benefits | 1.74 | (0.68-4.49) | 1.77 | (0.69-4.57) |
| 3 KMC benefits | 1.85 | (0.69-4.98) | 1.46 | (0.54-3.90) |
| 4 KMC benefits | 1.50 | (0.50-4.47) | 1.99 | (0.67-5.89) |
| 5+ KMC benefits | 1.27 | (0.33-4.91) | 1.65 | (0.47-5.75) |
| KMC Approval | 39.55** | (4.98-314.16) | 8.02*** | (2.88-22.35) |
| **Norms** |  |  |  |  |
| Descriptive norms | 4.96** | (2.12-11.63) | 1.54 | (0.75-3.17) |
| Normative expectations |  |  |  |  |
| Strongly agree | - |  | - |  |
| Agree | 0.06*** | (0.04-0.08) | 0.05*** | (0.04-0.07) |
| Strongly disagree/disagree | 0.07*** | (0.04-0.13) | 0.03*** | (0.02-0.06) |
| **Personal agency** |  |  |  |  |
| Self-efficacy index | 1.04** | (1.01-1.07) | 1.01 | (0.98-1.04) |
| KMC autonomy | 2.80*** | (1.62-4.84) | 0.71 | (0.45-1.14) |
| **Never Married** |  |  |  |  |
| No | - |  | - |  |
| Yes | 1.18 | (0.57-2.44) | 0.44 | (0.18-1.06) |
| **FTM’s highest level of schooling** |  |  |  |  |
| None/primary/secondary incomplete | - |  | - |  |
| Secondary complete or higher | 1.24 | (0.77-1.99) | 0.95 | (0.66-1.36) |
| **Household wealth** |  |  |  |  |
| Low | - |  | - |  |
| Medium | 1.04 | (0.69-1.58) | 0.66 | (0.42-1.02) |
| High | 0.89 | (0.57-1.40) | 0.84 | (0.55-1.29) |
| **Worked last year** |  |  |  |  |
| No | - |  | - |  |
| Yes | 0.95 | (0.64-1.41) | 0.93 | (0.66-1.31) |
| **Previous knowledge of KMC** |  |  |  |  |
| No | - |  | - |  |
| Yes | 1.27 | (0.66-2.44) | 0.98 | (0.55-1.74) |
| **Intervention health zone** |  |  |  |  |
| Comparison | - |  | - |  |
| Intervention | 1.08 | (0.76-1.55) | 1.12 | (0.80-1.58) |
| *Interaction term* |  |  |  |  |
| KMC autonomy*Never married | 0.75 | (0.32-1.74) | 2.85* | (1.06-7.63) |
| N | 926 | | 992 | |

*p<0.05, **p<0.01, ***p<0.001

Source: Momentum 2018 Baseline Survey and 2020 Endline Survey
