# Supplementary material for: The intention to use Kangaroo Mother Care among first-time mothers 15-24 years in Kinshasa, DRC: An application of the integrated behavioral model
Source: PLOS Glob Public Health. 2026 Oct 23;6(10):e0006015. doi: 10.1371/journal.pgph.0006015 (PMC13693924; doi:10.1371/journal.pgph.0006015)
Supplement: Supplementary file 3 [file pgph.0006015.s003.docx]

**Supplemental Table 3. Logistic regression models of KMC intentions among FTMs 15-24 years with previous knowledge of KMC, Kinshasa, DRC, 2020**

| **Variables** | **Model 1** | | **Model 2** | |
| --- | --- | --- | --- | --- |
|  | **Adjusted Odds Ratio** | **95% CI** | **Adjusted Odds Ratio** | **95% CI** |
| **Attitude** |  |  |  |  |
| Instrumental Attitude |  |  |  |  |
| No KMC benefits | - |  | - |  |
| 1 KMC benefit | 0.45 | (0.07-3.17) | 0.48 | (0.07-3.30) |
| 2 KMC benefits | 0.61 | (0.09-4.10) | 0.65 | (0.10-4.20) |
| 3 KMC benefits | 0.64 | (0.09-4.31) | 0.67 | (0.10-4.41) |
| 4 KMC benefits | 0.62 | (0.09-4.38) | 0.66 | (0.10-4.51) |
| 5+ KMC benefits | 0.50 | (0.07-3.75) | 0.54 | (0.07-3.98) |
| KMC Approval | 4.87** | (1.55-15.27) | 4.66** | (1.48-14.63) |
| **Norms** |  |  |  |  |
| Descriptive norms | 2.16* | (1.14-4.09) | 2.25* | (1.18-4.28) |
| Normative expectations |  |  |  |  |
| Strongly agree | - |  | - |  |
| Agree | 0.06*** | (0.04-0.08) | 0.06*** | (0.04-0.08) |
| Strongly disagree/disagree | 0.07*** | (0.04-0.14) | 0.07*** | (0.3-0.14) |
| **Personal agency** |  |  |  |  |
| Self-efficacy index | 1.00 | (1.00-1.03) | 1.00 | (0.97-1.03) |
| KMC autonomy | 1.30 | (0.84-2.00) | 1.66 | (0.79-3.46) |
| **Age group** |  |  |  |  |
| 15-19 | - |  | - |  |
| 20-24 | 0.70 | (0.47-1.04) | 0.86 | (0.39-1.93) |
| **Never Married** |  |  |  |  |
| No | - |  | - |  |
| Yes | 0.79 | (0.53-1.17) | 0.66 | (0.26-1.62) |
| **FTM’s highest level of schooling** |  |  |  |  |
| None/primary/secondary incomplete | - |  | - |  |
| Secondary complete or higher | 1.00 | (0.67-1.51) | 1.00 | (0.66-1.50) |
| **Household wealth** |  |  |  |  |
| Low | - |  | - |  |
| Medium | 0.88 | (0.57-1.37) | 0.88 | (0.57-1.37) |
| High | 0.75 | (0.47-1.17) | 0.74 | (0.47-1.17) |
| **Worked last year** |  |  |  |  |
| No | - |  | - |  |
| Yes | 0.88 | (0.61-1.27) | 0.89 | (0.61-1.29) |
| **Previous knowledge of KMC** |  |  |  |  |
| Yes | - |  | - |  |
| No | 1.14 | (0.63-2.07) | 1.13 | (0.63-2.06) |
| **Intervention health zone** |  |  |  |  |
| Comparison | - |  | - |  |
| Intervention | 1.00 | (0.70-1.43) | 1.00 | (0.70-1.42) |
| *Interaction terms* |  |  |  |  |
| KMC autonomy*Never married |  |  | 0.96 | (0.37-2.51) |
| KMC autonomy*Age |  |  | 0.64 | (0.27-1.53) |
| Age*Never married |  |  | 1.60 | (0.72-3.56) |
| N | 856 | | 856 | |

*p<0.05, **p<0.01, ***p<0.001

Model 1 and Model 2 also included household wealth, intervention or comparison health zone, employment in the last year, and previous knowledge of KMC

Source: Momentum 2018 Baseline Survey and 2020 Endline Survey
