# Supplementary material for: The intention to use Kangaroo Mother Care among first-time mothers 15-24 years in Kinshasa, DRC: An application of the integrated behavioral model
Source: PLOS Glob Public Health. 2026 Oct 23;6(10):e0006015. doi: 10.1371/journal.pgph.0006015 (PMC13693924; doi:10.1371/journal.pgph.0006015)
Supplement: Supplementary file 4 [file pgph.0006015.s004.docx]

**Supplemental Table 4. Firth’s penalized logistic regression model of KMC intentions among FTMs 15-24 years with previous knowledge of KMC, Kinshasa, DRC, 2020**

| **Variables** | **Model 1** | |
| --- | --- | --- |
|  |  | |
|  | **Firth Adjusted Odds Ratio** | **95% CI** |
| **Attitude** |  |  |
| Instrumental Attitudes |  |  |
| No KMC benefits | Ref |  |
| 1 KMC benefit | 1.56 | (0.77-3.14) |
| 2 KMC benefits | 1.85 | (0.96-3.57) |
| 3 KMC benefits | 1.65 | (0.83-3.28) |
| 4 KMC benefits | 1.79 | (0.84-3.81) |
| 5+ KMC benefits | 1.60 | (0.65-3.95) |
| KMC Approval | 10.94*** | (4.74-25.25) |
| **Norms** |  |  |
| Descriptive norms | 2.39** | (1.42-4.03) |
| Normative expectations |  |  |
| Strongly agree | Ref |  |
| Agree | 0.06*** | (0.04-0.07) |
| Strongly disagree/disagree | 0.05*** | (0.03-0.08) |
| **Personal agency** |  |  |
| Self-efficacy index | 1.02* | (1.00-1.05) |
| KMC autonomy | 1.42* | (1.06-1.90) |
| N | 1,918 |  |

*p<0.05, **p<0.01, ***p<0.001

Source: Momentum 2018 Baseline Survey and 2020 Endline Survey

Note: Regression was run with all demographic and study-context covariates included in the original multivariate logistic regression model; only theoretically specified predictors are shown in the above table
