# Supplementary material for: The intention to use Kangaroo Mother Care among first-time mothers 15-24 years in Kinshasa, DRC: An application of the integrated behavioral model
Source: PLOS Glob Public Health. 2026 Oct 23;6(10):e0006015. doi: 10.1371/journal.pgph.0006015 (PMC13693924; doi:10.1371/journal.pgph.0006015)
Supplement: Supplementary file 5 [file pgph.0006015.s005.docx]

**Supplemental Table 5. Logistic regression models using Webb wild cluster bootstrap of KMC intentions among FTMs 15-24 years with previous knowledge of KMC, Kinshasa, DRC, 2020**

| **Variables** | **Model 1** | | | |
| --- | --- | --- | --- | --- |
|  | **Adjusted Odds Ratio** | **95% CI** | **p-value** | **Webb bootstrap p-value** |
| **Attitude** |  |  |  |  |
| KMC Approval | 11.88*** | (5.01-28.12) | <0.001 | 0.01 |
| **Norms** |  |  |  |  |
| Descriptive norms | 2.44** | (1.44-4.13) | 0.001 | 0.03 |
| Normative expectations |  |  |  |  |
| Strongly agree | Ref |  | Ref |  |
| Agree | 0.05*** | (0.04-0.07) | <0.001 | 0.00 |
| Strongly disagree/disagree | 0.05*** | (0.03-0.08) | <0.001 | 0.01 |
| Joint Test of normative expectations |  |  |  | 0.004 |
| **Personal agency** |  |  |  |  |
| Self-efficacy index | 1.02* | (1.00-1.05) | 0.03 | 0.100 |
| KMC autonomy | 1.43* | (1.07-1.91) | 0.02 | 0.17 |
| N | 1,918 | | 1,918 | |

Source: Momentum 2018 Baseline Survey and 2020 Endline Survey

Note: Regression was run with all demographic and study-context covariates included in the original multivariate logistic regression model; only theoretically specified predictors are shown in the above table, instrumental variables are not included as they were not significant in the original model
